# Links between climatic histories and the rise and fall of a Pacific chiefdom

**DOI:** 10.1093/pnasnexus/pgae399

**Published:** 2024-10-01

**Authors:** Chuan-Chou Shen, Felicia Beardsley, Shou-Yeh Gong, Osamu Kataoka, Minoru Yoneda, Yusuke Yokoyama, Hsun-Ming Hu, Chun-Yuan Huang, Sze-Chieh Liu, Hong-Wei Chiang, Hsin-Lin Wei, Yun-Chuan Chung, Leilei Jiang, Albert Yu-Min Lin, James Fox, Mordain David, Jason Lebehn, Jason Barnabas, Gus Kohler, Zoe T Richards, Jean-Paul A Hobbs, Mark D McCoy

**Affiliations:** Department of Geosciences, High-Precision Mass Spectrometry and Environment Change Laboratory (HISPEC), National Taiwan University, Taipei 10617, Taiwan, ROC; Research Center for Future Earth, National Taiwan University, Taipei 10617, Taiwan, ROC; Anthropology Program, College of Arts and Sciences, University of La Verne, La Verne, CA 91750, USA; Department of Geology, National Museum of Natural Science, Taichung 40419, Taiwan, ROC; Department of Global Communication and Language, Kansai Gaidai University, Osaka 573-1001, Japan; Laboratory of Radiocarbon Dating, The University Museum, University of Tokyo, Tokyo 113-0033, Japan; Atmosphere and Ocean Research Institute, The University of Tokyo, Chiba 277-8564, Japan; Department of Earth and Planetary Science, Graduate School of Science, The University of Tokyo, Tokyo 113-0033, Japan; Graduate Program on Environmental Sciences, Graduate School of Arts and Sciences, The University of Tokyo, 3-8-1 Komaba, Meguro-ku, Tokyo 153-00419, Japan; Research School of Physics, The Australian National University, Canberra, ACT 0200, Australia; Department of Geosciences, High-Precision Mass Spectrometry and Environment Change Laboratory (HISPEC), National Taiwan University, Taipei 10617, Taiwan, ROC; Research Center for Future Earth, National Taiwan University, Taipei 10617, Taiwan, ROC; Department of Geosciences, High-Precision Mass Spectrometry and Environment Change Laboratory (HISPEC), National Taiwan University, Taipei 10617, Taiwan, ROC; Research Center for Future Earth, National Taiwan University, Taipei 10617, Taiwan, ROC; Department of Geosciences, National Taiwan University, Taipei 10617, Taiwan, ROC; Advanced Geological Research Task Force, Sinotech Engineering Consultants, Inc., Taipei 114065, Taiwan, ROC; Department of Geosciences, National Taiwan University, Taipei 10617, Taiwan, ROC; Department of Geosciences, High-Precision Mass Spectrometry and Environment Change Laboratory (HISPEC), National Taiwan University, Taipei 10617, Taiwan, ROC; Research Center for Future Earth, National Taiwan University, Taipei 10617, Taiwan, ROC; Department of Geosciences, High-Precision Mass Spectrometry and Environment Change Laboratory (HISPEC), National Taiwan University, Taipei 10617, Taiwan, ROC; Research Center for Future Earth, National Taiwan University, Taipei 10617, Taiwan, ROC; Department of Geosciences and Geography, University of Helsinki, 00014, Finland; Guangxi Laboratory on the Study of Coral Reefs in the South China Sea, Coral Reef Research Centre of China, School of Marine Sciences, Guangxi University, Nanning 530004, China; Qualcomm Institute, University of California, San Diego, CA 92093, USA; National Geographic Society, NW Washington, DC 20036, USA; Department of Chemistry, State University of New York, Stony Brook, NY 11794, USA; Pohnpei State Historic Preservation Office, Kolonia, Pohnpei 96941, Federated States of Micronesia; Pohnpei State Historic Preservation Office, Kolonia, Pohnpei 96941, Federated States of Micronesia; Pohnpei State Historic Preservation Office, Kolonia, Pohnpei 96941, Federated States of Micronesia; National Historic Preservation Program, Palikir, Pohnpei 96941, Federated States of Micronesia; Coral Conservation and Research Group, School of Molecular and Life Sciences, Curtin University, Bentley, WA 6102, Australia; Collections and Research Centre, Western Australian Museum, Welshpool, WA 6016, Australia; School of Biological Sciences, University of Queensland, Brisbane, QLD 4072, Australia; Department of Anthropology, Florida State University, Tallahassee, FL 32304, USA

**Keywords:** Saudeleur Dynasty, Nan Madol, coral ^230^Th dating, subsidence-induced sea level rise, ENSO

## Abstract

Sea level rise and climate change are shaping present societies, particularly those on oceanic islands. Few historical examples could serve as references for these changes. One such potential model is the Saudeleur Dynasty with its capital Nan Madol on the Pacific Island of Pohnpei. However, the timing of its construction, as well as the dynasty's fluctuations and potential environmental influences, has remained unresolved. Through the analyses of ^230^Th ages on 171 dates on corals fragments used as building materials and charcoal ^14^C ages from excavations, 2 major construction phases spanning from the 10th to the 15th century CE can be discerned. The results show that the first phase of the site's construction, spanning the 10th–12th century, marked the dynasty's rise. The second period, spanning from the late 12th to the early 15th century, provides the most substantial evidence for the demise of the island-scale chiefdom and a significant societal reorganization. The phases are centuries earlier than previously believed. With this new evidence, we propose the hypothesis that variations in the El Niño-Southern Oscillation and subsidence-related sea level rise presented major challenges for building and maintaining Nan Madol, and thus, influenced the course of the island's history. This case serves as a compelling example of how adverse climatic conditions can spur investments—in this case, in seawater defense under high sea levels—yet ultimately may contribute to abandonment. It offers lessons for island nations, showcasing coastal resilience in the face of worsening catastrophic events that unfolded over generations.

**Significance Statement**

Architectural coral ^230^Th dates and charcoal ^14^C ages reveal two major construction phases for Nan Madol, built with basalt boulders and coral rubble on the island Pohnpei in the Pacific Ocean, from the 10th to the 15th century CE. The first phase from the 10th to 12th century marked the dynasty's rise. The second from the late 12th to early 15th century marked its collapse. These phases were affected by variability of El Niño-Southern Oscillation and subsidence-related sea level rise. It imparts valuable lessons for our future, particularly for island nations, illustrating human resilience amid worsening climatic conditions for centuries.

## Introduction

A growing number of studies highlight the interplay between regional environmental histories and climatic conditions and the histories of human societies (1, 2). Hydrological cycles have, for example, been suggested to impact the rise and fall of ancient civilizations, such as those in Mesopotamia (3, 4) and across the Indian subcontinent (5). One of the most widely discussed events, the 4.2k year megadrought, may have strongly influenced the decline of civilizations in these regions and possibly beyond (6), while the social reorganization of the classic Maya, and abandonment of thriving urban centers with monumental architecture, has been attributed to a series of multidecadal drought conditions from 800 to 1,000 CE (7, 8). Still later, decades long extreme flood/drought events from the late 14th to early 15th centuries may have contributed to the demise of Khmer Empire in Cambodia leaving Greater Angkor unoccupied (9, 10).

One of the fundamental barriers to unpacking the historical relationship between climatic trends and ancient human societies is identifying when there is a plausible causal link. The inherent spatial and temporal limitations in the paleoenvironmental and archaeological records can lead us to over-determine or under-determine the role of the environment in human history, and mask the role of people in shaping and adapting to their environment (11). In this study, we explore how environmental variations—climate shifts, rising sea levels, and El Niño-Southern Oscillation (ENSO)—might have influenced the historical trajectory of the Saudeleur Dynasty, an island-wide chiefdom located on the Pacific Island of Pohnpei. Nan Madol (12, 13), the former capital the Saudeleur Dynasty, is central to unpacking how environmental changes may impact societal development.

**Fig. 1.**
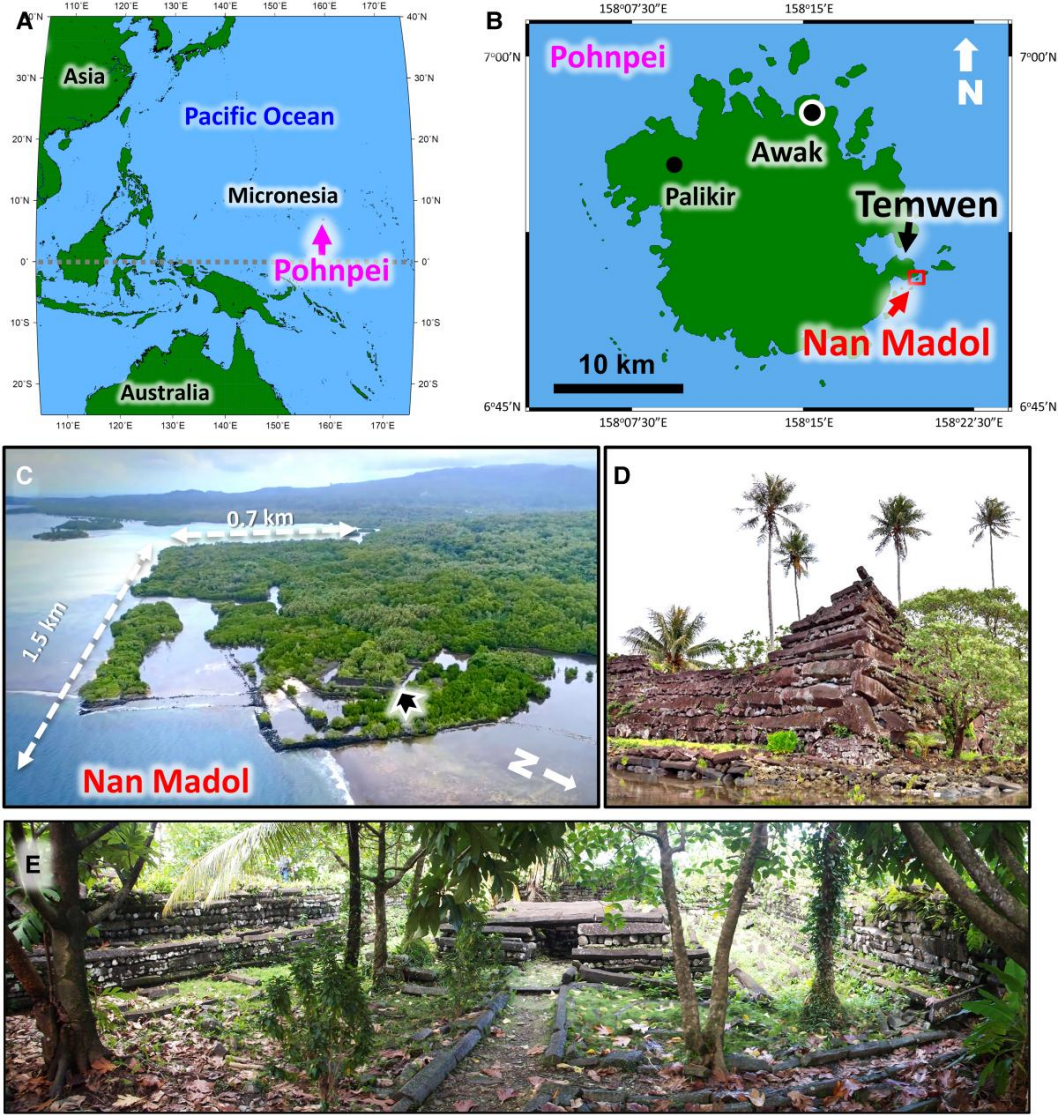
Maps of Pohnpei and Nan Madol. The ruins of Nan Madol and its artificial islets were built on the southeastern side of Pohnpei, Micronesia. A) Location of Pohnpei, the third largest island in Micronesia, in the northwest tropical Pacific Ocean. B) Map of Pohnpei showing the location of Nan Madol, capital of the Saudeleur Dynasty, located at the foot of Temwen Island. C) Aerial view of Nan Madol from the northeast. D) Northeastern corner of the outer wall of Nandowas islet (4–6 m in height, black arrow in C, location of the Royal Tomb Complex, built with columnar basalt and coral rubble). E) Central stone chamber at Nandowas where the Saudeleur chiefs were buried (photographed by Kataoka; courtesy of the National Museum of Ethnology, Osaka, Japan, used with permission). Maps in A and B were created with software Generic Mapping Tools Graphics v.5.1.1.

**Fig. 2.**
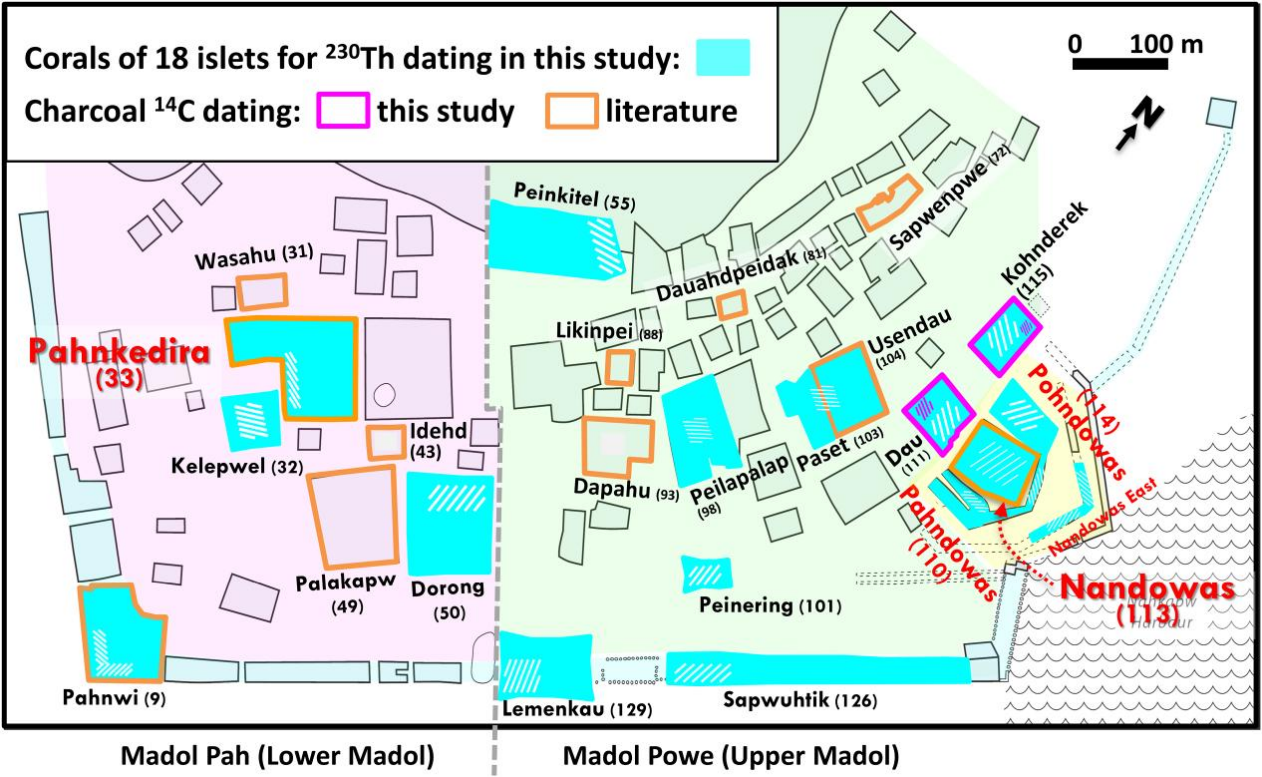
Nan Madol islets. Schematic of the site (modified from Ref (16)). Nan Madol is composed of two districts, Madol Pah and Madol Powe. In this study, Nan Madol is divided to four sectors of Central Madol Pah (light pink), Central Madol Powe (light green), Seawall Compounds (light blue), and the Royal Tomb Complex (yellow), consisting of Nandowas and the adjacent islets and compounds. Corals collected from 18 islets for ^230^Th dating in this study are indicated in cyan. Charcoal dates (Data S2) reported in this study from Dau and Kohnderek Islets are indicated with a fuchsia border, and those reported in literature (Data S3) are from islets with orange borders. Slashed areas represent the collection zones for the corals (white) and charcoal (fuchsia) reported in this study. Islets are identified by name with their associated number in brackets (after Ref (17)).

**Fig. 3.**
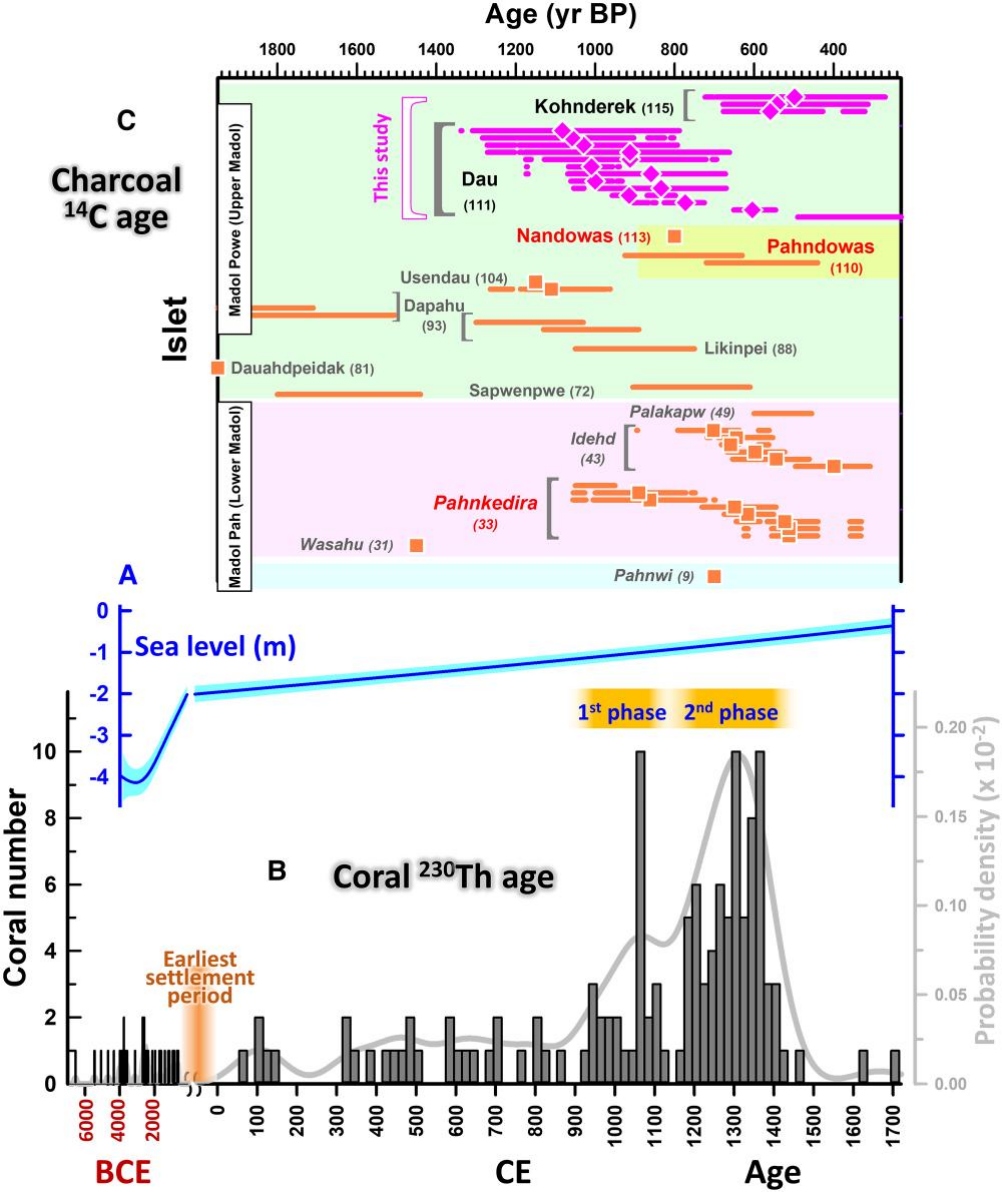
Relative sea level and distribution of coral ^230^Th and charcoal ^14^C ages. A) Pohnpei sea level curve (blue line) with a one-sigma uncertainty level (cyan shade) from BCE 3800 to CE 1700, relative to the condition in CE 2022 (16). B) 20-averaged histogram of coral ^230^Th ages from BCE 7000 to CE 1720 (Data S1), including four from Ref. (18) on Nandowas, from 18 islets across Nan Madol. Two corals with the same age of CE 1876 (Data S1) are not listed here. Gray line is the probability density curve, built with all coral ages and uncertainties. Orange bars denote the first and second phases of main construction stage. C) Calibrated charcoal ^14^C ages of islets from Central Madol Powe (light green), Central Madol Pah (light pink), Seawall Compounds (light blue), and Royal Tomb Complex (yellow). Ages for Dau and Kohnderek Islets (fuchsia) are reported here (Data S2) and others (orange) are from the literature (Data S3). Data presented with 2*σ* range intervals and median ages are IntCal20-calibrated (19); those with only points or ranges were reported in the literature with missing original measured ^14^C ages.

Built on the shoreline of Pohnpei, eastern Micronesia (Fig. 1A and B, Methods), Nan Madol is a monumental complex with megalithic architecture that at one time served as the capital of an island-wide chiefdom (Fig. 1C–E, Methods; Movie S1). Today it is comprised of >100 artificial islets separated by canals and monumental architecture built on top of islets (Figs. 1 and 2). An estimated 300,000 m^3^ of stone building materials and several tons of coral were used in the site's construction (12–14). Sefton et al. (15) used ^14^C-dated mangrove sediments to give a relative sea level rise model with a rate of ∼1 mm/year on Pohnpei since the middle Holocene (Fig. 3A). About 1,000 years ago, the entire site of Nan Madol may have sat on dry land, instead of its present condition with islets and canals.

A great deal of the history of Nan Madol remains only vaguely known. Chronologies for the site are almost entirely based in oral histories with little aid from material archaeological evidence. Few excavations have been conducted (20, 21). The results from these excavations were rarely adequately reported, and it is exceedingly rare that ^14^C dates from archaeological context meet contemporary standards of evidence (22). ^230^Th dating on coral that was live harvested as construction material for the central tomb on the islet of Nandowas, place its construction in CE 1180–1200, making it the earliest example of monumental architecture in the remote islands of the Pacific (18). Together these dates were used to argue that timing of Nan Madol, along with the rise and fall of the Saudeleur Dynasty, began around CE 1200–1300 and ended about CE 1500–1600 (23). Nunn (24) reviewed proxy records and proposed the possibility of climatic impacts on broader Pacific Island societies, although this remained a topic of debate (25–27). Despite over 150 years of scientific study, a chronology of Nan Madol, coupled with the potential climatic events that influenced its development, has yet to be established.

Coral ^230^Th dating techniques have been employed to understand the development of chiefdoms on Pacific islands (28–31). In this study, we present the construction history of Nan Madol based on high precision ^230^Th ages of corals used as building material across the site. These dates, along with ^14^C dating from excavations, suggest two major phases of construction between the early 10th and the early 15th centuries, and an intervening 40-year gap. The construction sequence of Nan Madol, we hypothesize, was influenced by environmental factors, specifically island subsidence-induced sea level rise and ENSO variability, and thus give us insight into an important, but understudied, link between climatic and social dynamics.

The megalithic monumental complex of Nan Madol is today located in the intertidal zone at the eastern foot of Temwen Island, Pohnpei, Micronesia (6°50′N, 158°19′E; Fig. 1A and B). It is a large village, mortuary, and religious complex that consists of over 100 large and small artificial islets constructed with basalt boulders and coral rubble (Fig. 1C and D), separated by navigable canals, and surrounded by a massive seawall. The site spans an area of 0.7 km (max. width) by 1.5 km (max. length) and is roughly rectangular in shape (Fig. 1C). Its islets range in size from 160 to 12,700 m^2^ (Fig. 2) (20).

The site is divided into two parts: northeastern Madol Powe (Upper Madol) with 60-plus islets and southwestern Madol Pah (Lower Madol) with over 30 islets (Fig. 2). We divided Nan Madol into four sectors: Central Madol Pah, Central Madol Powe, the Royal Tomb Complex, and the Seawall Compounds (Fig. 2). The most important islet in Central Madol Pah is Pahnkedira, where the paramount chiefs of the Saudeleur Dynasty resided and exercised their power (14). Central Madol Powe supports the priestly residences and sacred spaces that served the Saudeleurs (13). The Royal Tomb Complex, at the eastern extremity of Nan Madol, was reserved for the Saudeleurs and encompasses Nandowas Islet (Fig. 1D and E), a ritual and burial center that served as a fort during times of war (12–14). The Seawall Compounds, including Pahnwi Islet, formed the breakwater from the earliest stages of site occupation (13).

Eighteen islets across of the main four sectors, including Central Madol Pah, Central Madol Powe, Seawall Compounds, and the Royal Tomb Complex (Fig. 2), of Nan Madol were sampled for corals in 2012, 2016, and 2018 (Figs. S1–S6). In all, 167 specimens of 172 corals were selected from islets for ^230^Th dating (32) (Figs. S1–S6). Combined with four ^230^Th dates from McCoy et al. (18), a total of 171 coral dates, from the middle Holocene to the 19th century, were listed in Data S1a and plotted in Fig. 3. We also report additional 18 calibrated charcoal ^14^C dates, ranging from the 9th to 15th century, from layers with artifacts in the 2005 excavations on the islets of Dau and Kohnderek (Figs. S5 and S6). We stipulate that ^14^C dates on unidentified plant charcoal, both previous dates and the dates presented here, are known to be problematic as a chronometric tool in archaeology in the Pacific (33). We include them here to give a full picture of all relevant data on the construction of Nan Madol.

## Results and discussion

### Coral and charcoal ages

The overall distribution of coral dates ranges from 6552 BCE to CE 1873 (Fig. 3B) and includes both corals harvested live for building material and naturally accumulated coral fossil rubble that would have been gathered from the shoreline or nearshore. Archaeological and linguistic studies suggested the eastern end of the Caroline Islands, including Pohnpei, was colonized around 500–1 BCE (13). Samples dating back to 6552–614 BCE, predating human presence in the region (13), are therefore considered to be coral fossil rubble (*n* = 40, 0.13 per 20 years). The absence of any corals dated between 614 BCE and CE 69 implies that the first Pohnpeian inhabitants might not have built compounds at the site. From CE 69 to 900, there are 28 dated samples. The appearance of coral with a rate of 0.67 per 20 years is higher than before. They could be classified as fossils. These corals could represent the more accessible, recent surface coral rubble that was gathered more easily when construction began. They could be also live-harvested corals used for the possible initial construction, based on charcoal ages from 0 BCE to CE 510 (22, 23) (Fig. 3C).

**Fig. 4.**
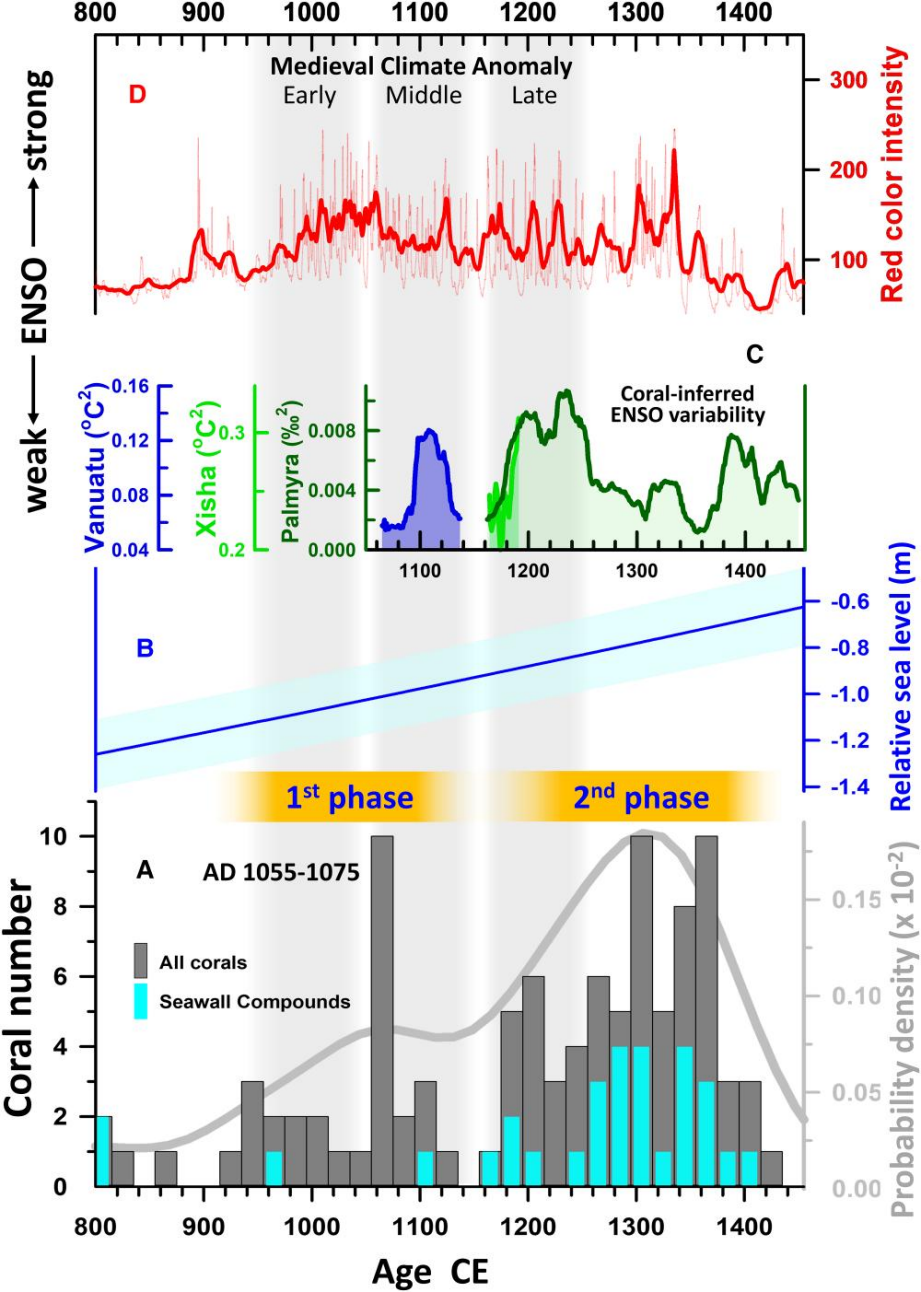
Distribution of corals in Nan Madol, Pohnpei sea level, and ENSO variability records between CE 800 and 1455. A) 20-averaged histogram of coral ^230^Th ages. All corals (Data S1) are colored in dark gray. Corals from the Seawall Compounds are colored in cyan. Gray line is the probability density curve. Orange bars denote the 1st and 2nd phases of main construction stage. A 20-year peak at CE 1055–1075 at the first phase is highlighted (see text for details). B) Pohnpei sea level (blue line) is shown at a one-sigma uncertainty level (cyan shade) relative to the conditions in CE 2022 (15). Qualitative ENSO variability inferred from (D) variances in a sliding 30-year window for the filtered coral Sr/Ca-sea surface temperature (SST) anomalies (CE 1050–1151) from Vanuatu (34) (blue), Sr/Ca-SST anomaly (CE 1149–1205) from Xisha Islands (35) (green), and δ^18^O anomaly (CE 1149–1220) from Palmyra Island (36) (olive green), and D) 30-year average curve (think red line) of sediment red color intensity records from lake Laguna Pallcacocha, Ecuador (37). The thin red line represents raw annual data. It is important to note that “the absolute intensity of red color and the width of the individual laminae do not correspond to the intensity of the ENSO event” (37). All records are given with original age models with 2*σ* errors of ± 4–5 years for coral records and of ± 5% for sediment records. The relative ENSO variability is depicted with arrows for records in C and D. Three light gray vertical bars respectively denote the early, middle, and late periods of the MCA (∼CE 950–1250).

The probability density curve, built with all coral ^230^Th ages and uncertainties, shows an abrupt increase of coral numbers after CE 900 and two peak domains between the early 10th and the early 15th century (Fig. 3B). A coral use rate of 3.9 per 20 years (*n* = 98) for 500 years (CE 930–1430) is dramatically higher than 0.13 and 0.67 per 20 years in the previous time intervals. It indicates that harvests for construction mainly occurred during this period. The date range is consistent with our new ^14^C dates on two islets of Dau and Kohnderek and previously reports, with medians from the 9th to 15th century (Fig. 3C). This consistency suggests that the majority of the corals were collected alive after CE 900, with even the potential inclusion of fossil corals cannot be ruled out. The coral ^230^Th ages reveal the first early phase from the early 10th to the early 12th century and second late one the late 12th to the early 15th century, separated by a 40 years gap (Fig. 4A). Five samples were dated during the early 15th to 19th century (CE 1463–1876; Fig. 3B and Data S1), after the second main construction phase.

### Before the early phase building

The Island of Pohnpei has subsided by 4 m since the mid-Holocene (15) (Fig. 3A). During the earliest settlement period, 500–1 BCE (13), the island was still 2.0–2.5 m higher than present. The current artificial channels between Nan Madol's islets have filled with silt deposits anywhere from 10 to 100 cm in depth (21). The averaged water depth of channels is 60 cm at middle tide based on our observations in our field surveys in CE 2016 and 2018 and previous reports (38). These lines of evidence suggest that the eastern coast of Temwen Island was a beach roughly 0.4–1.8 m higher than sea level when the founding population of Pohnpei arrived (13). This inference is supported by the excavations in 2018 by Sand et al. (21), indicating the original substrate of Nan Madol consisted of sand, coral rubble, and fragmented shells.

The tropical Pacific climate is dominated by ENSO variability (39). Intensified ENSO variability and the associated strong La Niña patterns (36) result in heightened prevailing easterly wind surges (40), increased windward rainfall (41), and the onset of wave intrusion and erosion in the central and western Pacific, as noted by Vos et al. (42). In the case of Pohnpei Island from CE 1978 to 2020, the regional sea level can be elevated by 10–20 cm during La Niña events, or even over 30 cm for 10 days in September of 1988, a strong La Niña year (see Fig. S7). Nunn et al. (43) have documented that sea level rise has the potential to induce shoreline erosion along the windward coast. Consequently, the rising sea level, coupled with intensified wind surges and windward precipitation, may lead to accelerated shoreline erosion. The climatic and environmental changes could affect the inhabitants' way of life and necessitating the construction of protective barriers.

Sediment records at Laguna Pallcacocha in Ecuador (37), located in ENSO climate zone, indicate an increasing ENSO variability across the Pacific Basin from the mid-Holocene. The remains of biotic carbonate debris on Temwen beach before 2000 years ago (21) could have been delivered by strong easterly winds during prevailing La Niña years under a period with high ENSO variability.

Between the arrival time of the earliest people (13) and CE 900, Pohnpei's elevation was ∼2.4–1.2 m higher than its current level (Fig. 3A). Pohnpei experienced a nearly 1,000-year period of weak ENSO activity, except for one 70-year and one 130-year interval at CE 300 and 610, respectively (Fig. 1B of Ref. (33)). The presence of a coast characterized by relatively high terrain and abundant carbonate debris substrate (21), combined with minimal wave action due to the weak easterly winds resulting from reduced ENSO variability, would likely have been among the influencing factors that led to the selection of the location for Nan Madol.

### The early phase building

Our discovery of a previously undocumented early phase of construction from the early 10th to the early 12th century (∼CE 930–1130; Fig. 4A) is essential. The dates of 27 corals (Data S1) in this phase are from locations across the site. This finding suggests that the commencement of monument construction likely occurred during the early 10th century. A single prominent 20-year peak at CE 1055–1075 is observed by 10 dated corals (35% of all corals used in the phase) from the palace Pahnkedira, where the paramount chiefs of the Saudeleur Dynasty resided and exercised their power, and the Royal Tomb Complex, including Nandowas, Pahndowas, and Pohndowas (Fig. 2). Such a concentration of activity argues for the establishment of a new ruling class through the appearance of the Dynasty (44–46). This new evidence suggest the Saudeleur Dynasty may have been established much earlier than previously thought (at CE 1180–1200, (18)).

Before the commencement of the early phase of construction, this site may have experienced weak easterly winds resulting from less intense La Niña events under the subdued ENSO variability (Fig. 4C and D). Pohnpei was about 1.2 m higher than present (Fig. 4B). The evidence suggests that when the main construction of the early phase began in the early 10th century (Fig. 4A), the site could have been exposed to fewer natural hazards, resulting in a more favorable environment for construction. The climatic and hydrodynamic conditions changed dramatically since the early Medieval Climate Anomaly (MCA), supported by 30-year sliding coral-inferred ENSO variance records from central (36), southwestern (34), and western Pacific (35) (Fig. 4C), as well as lake sediment red color intensity records at Laguna Pallcacocha in Ecuador (37), southern America (Fig. 4D). These records suggest Nan Madol could have experienced regular wave intrusions and increased siltation at high tides during the middle MCA, until a brief reversal to mitigated ENSO conditions at the end of middle MCA (Fig. 4).

### Transition from early to late phases

During the middle to late MCA in the mid-12th century, only one architectural coral was discovered in Pahnkedira (Fig. 4A and Data S1b). This period, spanning 40 years, was characterized by diminished ENSO variability (Fig. 4C and D). The results suggest that a dramatic decrease in the construction activities at Nan Madol was linked to a relative calm climatic condition. Severe tidal surges during this period would have subsided, wind and current patterns would have weakened, and wave action may not have breached or posed a major threat to the site compounds.

### The late phase building

The late phase from the late 12th to the early 15th century (∼CE 1170–1411, or −1425) saw accelerated construction with 69 (or 70) dated corals representing 70% of corals since the first phase construction (Data S1b). The major complement of ^14^C dates fits well within this interval (Fig. 3C). The evidence suggests that construction activities across the site were intensified. During this phase, the seven dated corals from Pahnkedira, for example, represent almost double the number of dated corals from the previous phase. The density of coral and charcoal dates in this period (Fig. 3) supports increased levels of activity, as well as elevation in the importance of Nan Madol as a central place—this was the most powerful period in the Dynasty.

The significant increase in coral use, averaging about 5.6 per 20 years during this phase, more than doubled compared with the first phase. This suggests a high level of construction activity (Fig. 4A). ENSO variability intensified around CE 1180–1250 in coral proxy records (Fig. 4C) and from the late MCA to CE 1340 in sediment records (Fig. 4D), followed by a subsequent decrease. The histogram of coral ^230^Th ages reveals a notable peak spanning 20 years with 10 dated corals between CE 1295 and 1315, and another peak spanning 40 years with 18 corals between CE 1335 and 1375, indicating a discrepancy with the intensified ENSO variability window. During this phase, the coral usage history diverges from the ENSO variability trend (Fig. 4A), suggesting that ENSO was not the main explanatory factor at this time.

Compared with the relatively low sea level of −126 cm at CE 800, the sea level rose, reaching −90 and −70 cm at CE 1180 and 1380, respectively (Fig. 4B). Taking into account silt deposits averaging 10–100 cm in thickness (21) and the average modern water depth of 60 cm in channels, by CE 1180, channels were either dry or submerged under seawater up to 70 cm at middle tide. The situation deteriorated further, with seawater intruding into all channels by around CE 1380. The relatively high sea level during this second phase would have breached the seawall, exacerbating damage to the site compounds and increasing the need to accelerate construction/reconstruction activities across the site. Reinforcement of the seawall compounds would have been an immediate and important focus of construction activity (Fig. 4A) to protect the site from further inundation, damaging tidal surges, and strong wave action. This observation is supported by the finding that up to the maximum 37 or 38% (26/69 or 26/70) of dated corals from this second phase occur in the Seawall Compounds, remarkably larger than only 7% (2/26) in the first early phase (Fig. 4A and Data S1b).

The site was largely abandoned in the early 15th century. There is a drop off in evidence of coral usage for architecture that suggests a main stoppage of construction work, including maintenance activities, repairs, and renovations. Such cessation of large-scale construction at Nan Madol likely presages an end to the Saudeleurs and another shift in the sociopolitical system of Pohnpei. This end date for Nan Madol does not mean its full abandonment as sporadic construction or renovations likely occurred because the site continued to serve as the seat of governance for the first Nahnmwarki and his initial successors (13, 44).

### Postabandonment reconstruction

Five corals dated to after the abandonment of Nan Madol as the island's capital (Data S1b), one (CE 1463) from Pahnwi of the Seawall Compounds, two (CE 1634, and 1704) from the Central Madol Powe, and two from Nandowas with the same latest age of CE 1876 (Data S1b). The ages indicate that corals were sporadically used several times from the middle 15th to 19th century. It is not surprising that people would organize to repair and revitalize this site given its importance. While these postdate European contact, they are both during periods when there is an absence of colonial rule of the island (47).

## Conclusions and perspectives

We analyzed 167 coral ^230^Th ages from 18 islets and 18 charcoal ^14^C ages of 2 islets. Combined with previous coral and charcoal ages, the results express two major phases of construction between the early 10th and the early 15th centuries, associated with rise and fall of the Saudeleur Dynasty. The history of the chiefdom is likely shaped by a blend of intertwined natural and social forces, making it difficult to establish a direct statistical linkage between climate factors and the construction of Nan Madol. However, the distribution of coral ages can still provide temporal evidence that potentially links subsidence/ENSO events to the construction sequence. In particular, the steady rise in sea level during the second phase would have necessitated increasingly large and frequent protective efforts, likely leading to enhanced construction of sea walls. Further examination of more corals from islets at this site could offer deeper insights into the detailed construction history.

Dating results reveals that the history of construction at Nan Madol reflects a people drawn into a cycle of repair and investment into protections from future coastal disasters. ENSO events, which would have brought episodic damages, and subsidence, which would have made slow incremental damage, for centuries were met with resilience rather than abandonment or social reorganization. These same forces may have eventually contributed to the end of the island-scale chiefdom and a halt to new construction at its capital.

What drew people into this cycle of construction and reconstruction? One popular, but untested, hypotheses suggests that new breeds of breadfruit (*Artocarpus altilis*) increased local food production capacity and that had knock on effects for human population growth and labor available for large construction projects (48). The local capacity for agricultural surplus, therefore, may have been a key factor. The connection between the timing of dynasty's rise and fall, associated with Nan Madol construction, and changes in tectonic displacement and climatic conditions over the MCA could articulate with the hypothesized “breadfruit revolution.” The halt in constructions in the early 15th century follows the commencement of the Little Ice Age. Nunn et al. (49) argued that following the MCA, a “massive and rapid reduction of the food resource base” during the AD 1300 event (24), could have induced “social disruption” for communities dispersed across the Pacific. It also, however, remains untested due to the lack of relevant datasets regarding the distribution of human settlement across the island and variations in food production.

The persistent intersection of climate impacts and construction activity highlights an ongoing necessity for the maintenance of large-scale structures that are susceptible to regular damage from natural hazards. This continuous need for repairs has given rise to a cycle in which damage acts as a “catalyst.” This cyclical pattern could account for the 40-year gap in construction evidence between the early and late phases, which aligns with a period of reduced ENSO activity.

In the late phase, coral ages show even greater investments in monuments, with more emphasis on sea breaks as sea levels rose, and the transport of columnar basalt from a great distance away from the site (16). Oral histories point to the high demands of the Saudeleur as the main cause for their overthrow, and a return to district-scaled political authority. Our results are consistent with construction efforts being at an all-time high at the time the site was abandoned. It may have been the case that the Saudeleur's continued demands for labor, in the absence of damage to justify the effort, over time recast these once regular tasks into an unreasonable burden on the people. This underscores the importance of considering the archaeological record as an archive of human societal experiments and resilience (50).

This case gives us a long-term antecedent model for the challenges island communities around the world face through climate change. Under the recent 3-year-long La Niña period from CE 2020–2022, for example, seawater invasion hit numerous villages of sinking islands, such as Solomon Islands (Fig. S8), Papua New Guinea (Movie S2), and Cook Islands (Movie S3) over the Pacific Ocean. As a mirror on the possible fate of island lifeways, our study stands as a prescient warning. With the current intensification of variabilities of ENSO (51) in the Pacific Ocean and its counterpart, Indian Ocean Dipole, in the Indian Ocean (52) along with sea level rise (53) exceeding 3 mm/year, the coming decades will likely experience the inundation of more islands and an increase in the numbers of climate refugees (54, 55). The case of Nan Madol raises the question of whether ongoing climate change will lead to the abandonment of coastal and oceanic communities, or prompt investment in local infrastructure for climate migitation.

## Methods

### Site description

Islet platforms of Nan Madol are paved with basalt boulders and coral gravel (Figs. 1E and S1). Most of the islets are covered by a dense growth of vegetation, with expansion of the mangrove ecosystems engulfing the nearshore islets and exacerbating siltation of the canals. Extensive root systems from the encroaching vegetation have worked their way into islet foundations, loosening walls and compromising the architectural integrity of these structures, further increasing their fragility. Nan Madol was built over the course of centuries and served as home to the leaders of the Saudeleur Dynasty that controlled the ritual cycle and political administration of Pohnpei. The rise and fall of the Dynasty parallel the history of Nan Madol.

Nan Madol was inscribed onto UNESCO's World Heritage List in 2016 and at the same time placed on the list of World Heritage in Danger owing to ongoing deleterious effects of an aggressive climate change. The site played a key role in influencing the direction and growth of the socioeconomic–political complexity throughout the region.

### Field sampling

Coral samples were drawn from the core fill of architectural features across the site, with samples extracted from breaches within these features. Each sampling location was identified with GPS coordinates, as well as triangulated onto a plan map of the site. Anywhere from 1 to 17 coral samples were collected from each of the selected islets (Figs. 2 and S1; Data S1). See McCoy et al. (18) for field sampling of samples collected in 2012. The overall sampling method was relatively noninvasive from areas where it was unlikely the coral fill would have been replaced during routine maintenance. Although at some point during the long history of site occupation, the core coral fill could have been augmented through maintenance, reconstruction, or renovation activities. We selected only the corals with undamaged corallite appearances. Corals attached to reef rocks used on islets were most likely quaternary fossils and were avoided. Once collected, each coral sample was identified to genus level.

The sampling process was undertaken during extended periods of low tide, with each of 18 islets selected based on their location within the site and their historical roles. All islets were approached on foot by traversing the canals between islets, many of which were choked with sediment and mangroves. Access to the outer perimeter/seawall was by way of the main canal running through the site and exiting onto the shallow reef platform on which Nan Madol is built.

For charcoal ^14^C dating, six test pits (TP-1 to TP-6) were excavated on Dau (Fig. S5) and two test pits (TP-1 and TP-2) on Kohnderek (Fig. S6). Eighteen samples were recovered during these excavations, conducted between July 25 and August 20, 2005.

### Coral ^230^Th dating

In total, 172 subsamples, each 3–10 g, were chipped on-site from the coral samples collected across the site (Figs. S1–S6). X-ray diffractometry was used to confirm the pristine qualities of each coral sample—5 of the corals were determined to be altered and subsequently removed from the analysis, while 167 corals with an aragonitic composition were considered pristine and selected for ^230^Th dating. A small segment of each sample, 5–10 mm^3^, was carefully cut from the third annual band from the top edge of each subsample. This segment was gently crushed into 0.3–1 mm^3^ pieces and physically cleaned with ultrasonic methods (56). About 100–200 mg was used for chemistry (57) and instrumental analysis (32, 58). Isotopic compositions and concentrations were determined on a multicollector inductively coupled plasma mass spectrometer, Thermo Neptune, in the High-Precision Mass Spectrometry and Environment Change Laboratory (HISPEC), Department of Geosciences, National Taiwan University (32). U-Th isotopic measurements and ^230^Th age results are summarized in Data S1. Dating criteria applied to evaluate reliable ages for the pristine corals were based on a value of 146 ± 8 for the initial δ^234^U and U contents of 1.5–4.0 ppm (59, 60). The half-life values used in age calculation are listed in Cheng et al. (61). Coral death dates were calculated by subtracting 3 (±1) years from the corrected ^230^Th ages. Results of 167 coral ^230^Th dates and 5 previously published ^230^Th ages from Nandowas (18) are listed in Data S1. A date of CE 809 with large uncertainty of ±275 years for one coral (C-19) in McCoy et al. (18) is excluded. The number of coral dates used in this study is 171.

### Charcoal ^14^C dating

Fifteen charcoal samples, 721 g in total weight, were collected from layers with artifacts during the Dau excavations (Fig. S5) and three samples, 295 g in total weight, collected on Kohnderek (Fig. S6). Prior to dating, all samples were examined for integrity, and subjected to physical and chemical pretreatment procedures to remove contaminants such as rootlets. Physical pretreatment methods involve visual inspection of samples and the removal of secondary carbon contributions (e.g. rootlets); chemical methods employ the standard acid–alkali–acid method to further remove impurities. Cleaned samples were dated at the Micro Analysis Laboratory, Tandem Accelerator, University of Tokyo, and the Tandem Accelerator for Environmental Research and Radiocarbon Analysis at the National Institute for Environmental Studies (62). Two of the Dau samples could not be dated; these were collected from the 0–10 cm level of TP-4 and 130–140 cm level of TP-6. Calibrated dates and data for 16 charcoal samples from both Dau and Kohnderek Islets are recorded in Data S2. Ages are calculated at 2-sigma intervals (InCal20, Rev 8.2) (19). Previously published charcoal ages are given in Data S3.

## Supplementary Material

pgae399_Supplementary_Data

## Data Availability

All data are available in the main text or the Supplementary material.
