# Supplementary material for: Links between climatic histories and the rise and fall of a Pacific chiefdom
Source: PNAS Nexus. 2024 Oct 1;3(10):pgae399. doi: 10.1093/pnasnexus/pgae399 (PMC11443547; doi:10.1093/pnasnexus/pgae399)
Supplement: pgae399_Supplementary_Data [file pgae399_supplementary_data.zip › Shen et al. Nan Madol Supplementary material-final.pdf]

PNAS nexus

Supplementary material for

Links between climatic histories and the rise and fall of a Pacific chiefdom

Chuan-Chou Shen*, Felicia Beardsley, Shou-Yeh Gong, Osamu Kataoka, Minoru Yoneda, Yusuke Yokoyama, Hsun-Ming Hu, Chun-Yuan Huang, Sze-Chieh Liu, Hong-Wei Chiang, Hsin-Lin Wei, Yun-Chuan Chung, Leilei Jiang, Albert Yu-Min Lin, James Fox, Mordain David, Jason Lebehn, Jason Barnabas, Gus Kohler, Zoe T. Richards, Jean-Paul A. Hobbs, Mark D. McCoy

*Corresponding author: Chuan-Chou Shen
Email: river@ntu.edu.tw

This PDF file includes Figures S1 to S8, titles of Data S1 to S3, and titles of Movies S1 to S3.

Other supporting materials for this manuscript include the following:

Data S1: Coral U-Th table and ^{230}Th ages
Data S2: Charcoal ^{14}C dates on Dau and Kohnderek
Data S3: Charcoal ^{14}C dates from the published literature
Movie S1: Nan Madol
Movie S2: Seawater invasion to Carterets, Papua New Guinea on December 6, 2021. (Courtesy of the Bougainville Today, used with permission.)
Movie S3: Seawater invasion to Rarotonga, Cook Islands on July 15, 2022. (Courtesy of Ahmad Shodiq, used with permission.)

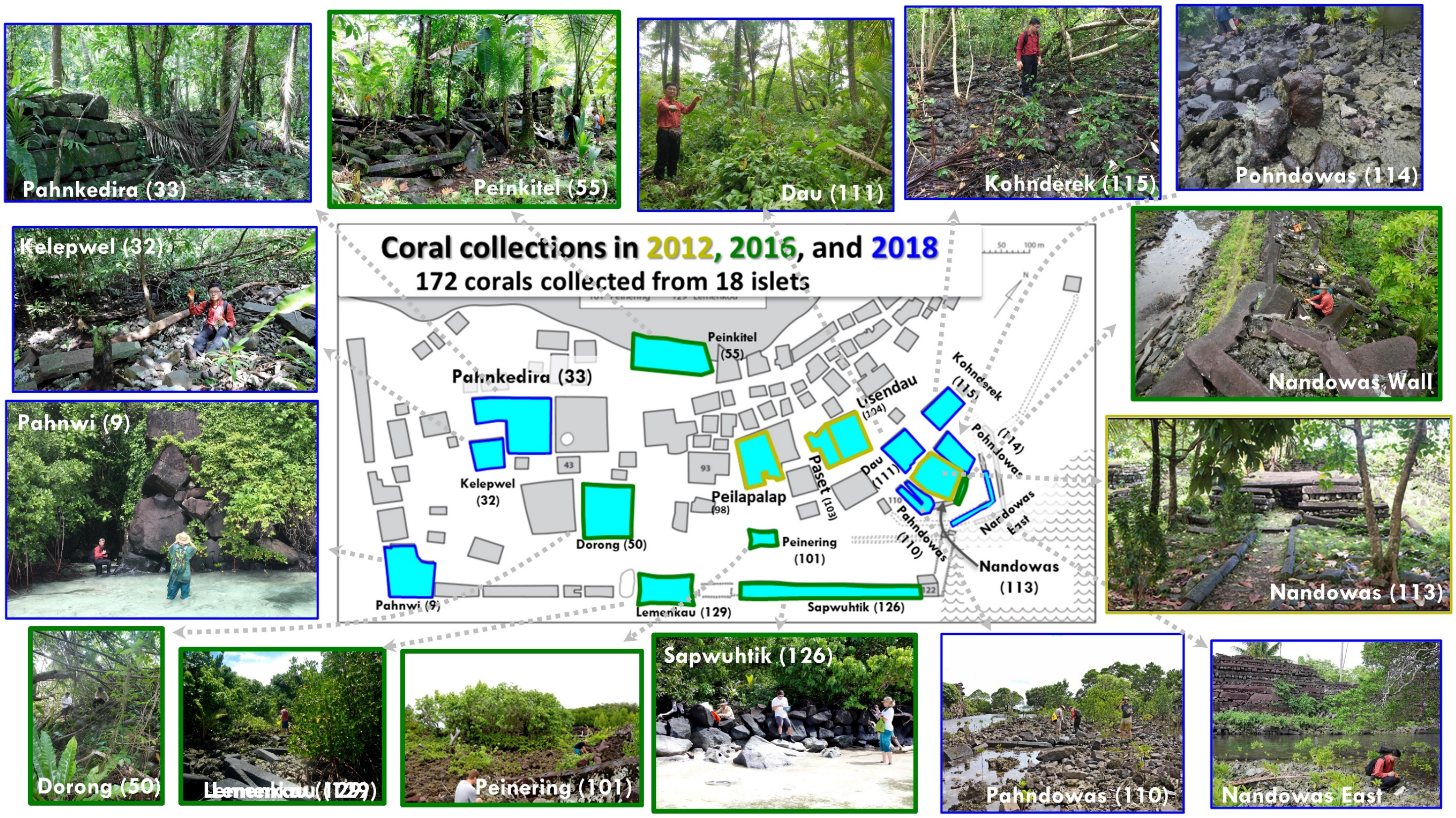

Fig. S1. One hundred and seventy-two corals collected from 18 islets in 2012, 2016, and 2018 in this study. Photographs of only 15 islets are given here as examples. Islets in cyan on the map and the corresponding photographs with a dark yellow border dark for the collection in 2012 (ref. 18), a green border in 2016, and a blue border in 2018. Islets are identified by name with their associated number in brackets (after ref. 17).

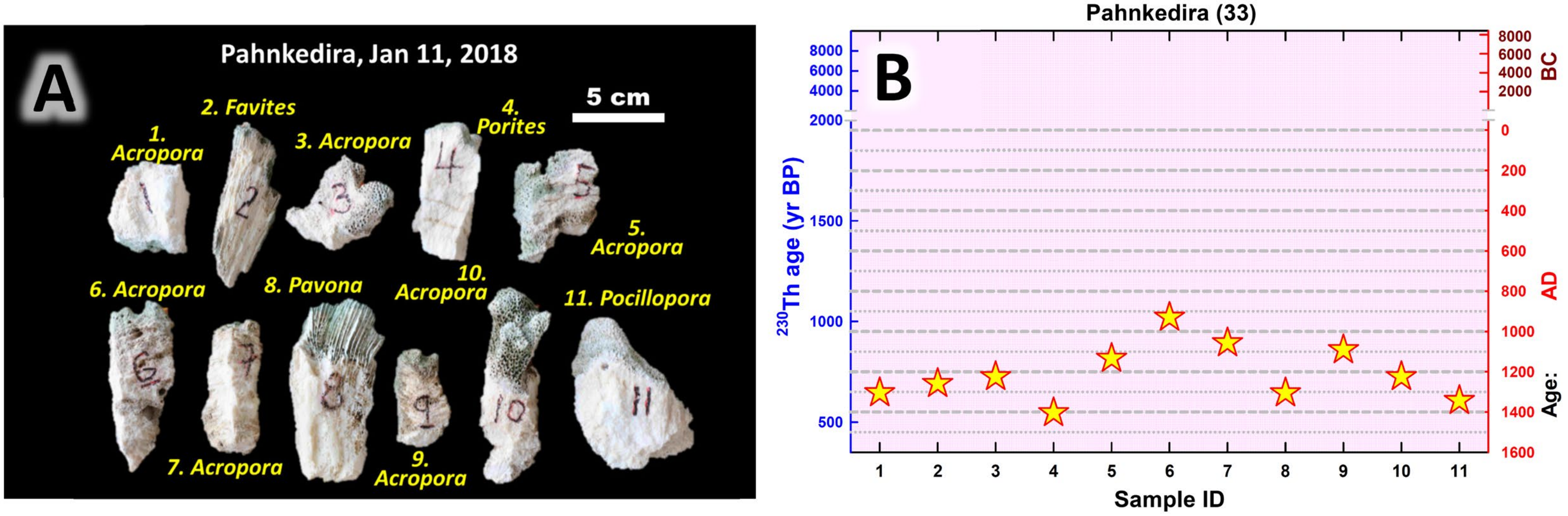

Fig. S2. Corals collected from Pahnkedira Islet. (A) Eleven coral subsamples were collected in 2018. **(B)** The determined ^{230}Th ages.

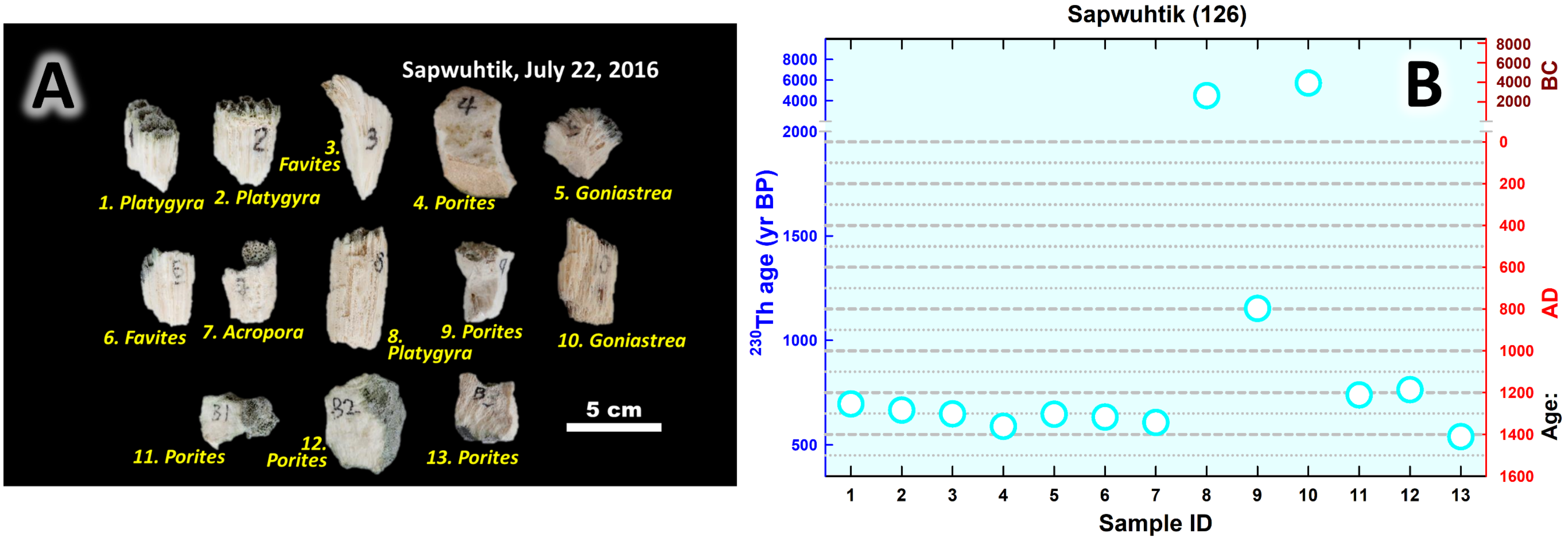

Fig. S3. Corals collected from Sapwuhtik Islet. (A) Thirteen coral subsamples were collected in 2016. **(B)** The determined ^{230}Th ages.

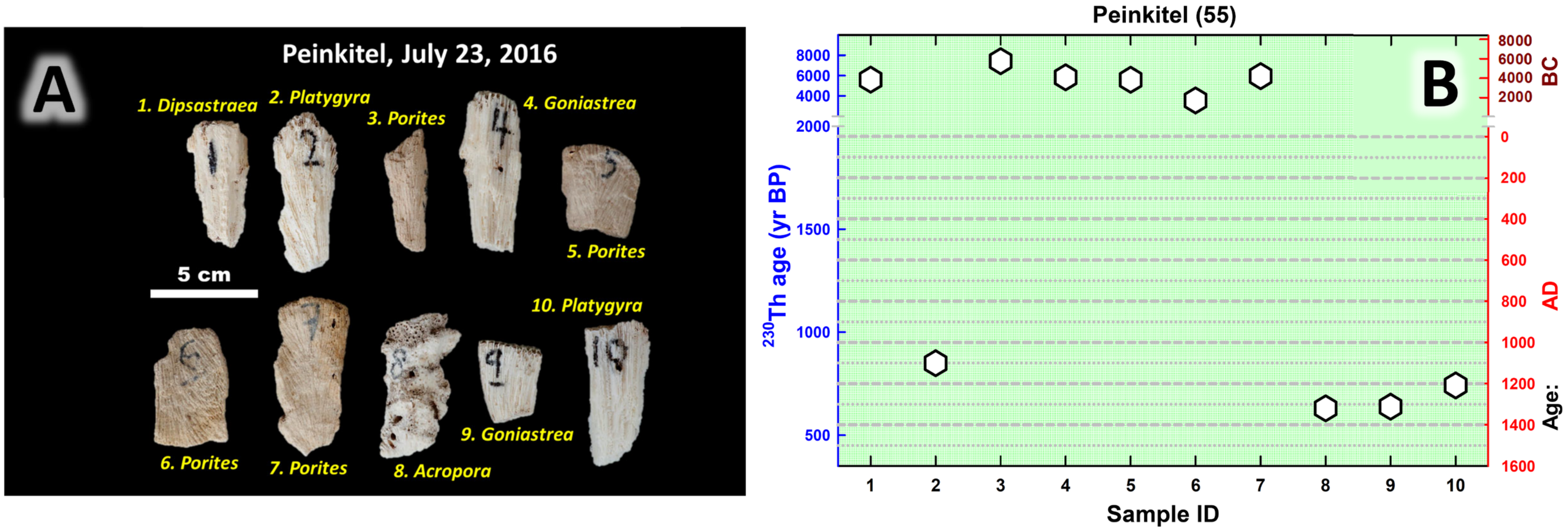

Fig. S4. Corals collected from Peinkitel Islet. (A) Ten coral subsamples were collected in 2016. **(B)** The determined ^{230}Th ages.

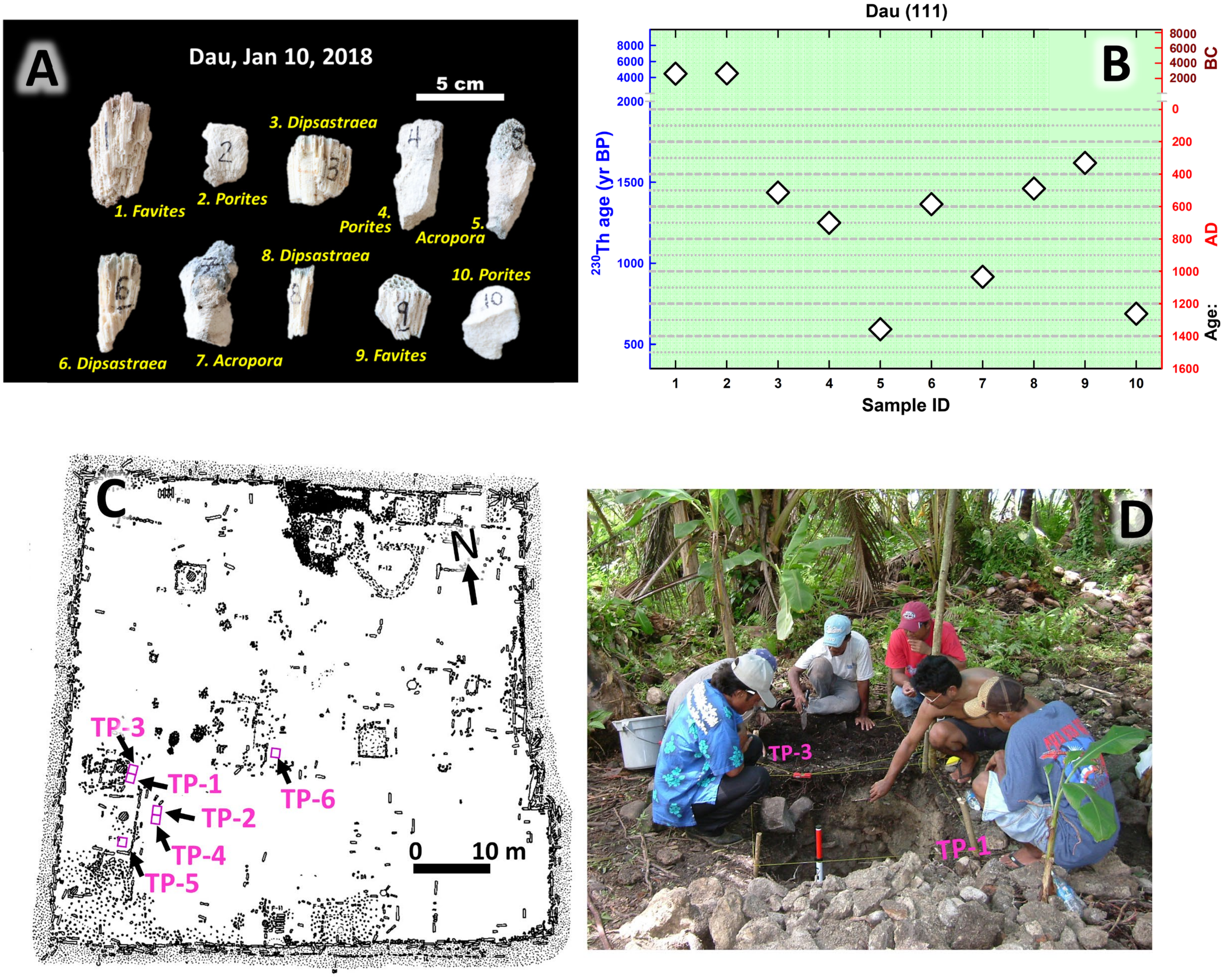

Fig. S5. Corals and charcoals collected from Dau Islet. (A) Ten coral subsamples were collected in 2018. **(B)** The determined coral ^{230}Th ages. **(C)** Six 1x1 m test pits were excavated on Dau (map modified from ref. 63). Charcoal samples of TP-1, TP-2, TP-5, and TP-6, were collected during August 8-20, 2005, for ^{14}C dating (Data S2). **(D)** A photograph of test pits TP-1 and TP-31 (taken by Kataoka in 2005; courtesy of the National Museum of Ethnology, Osaka, Japan, used with permission).

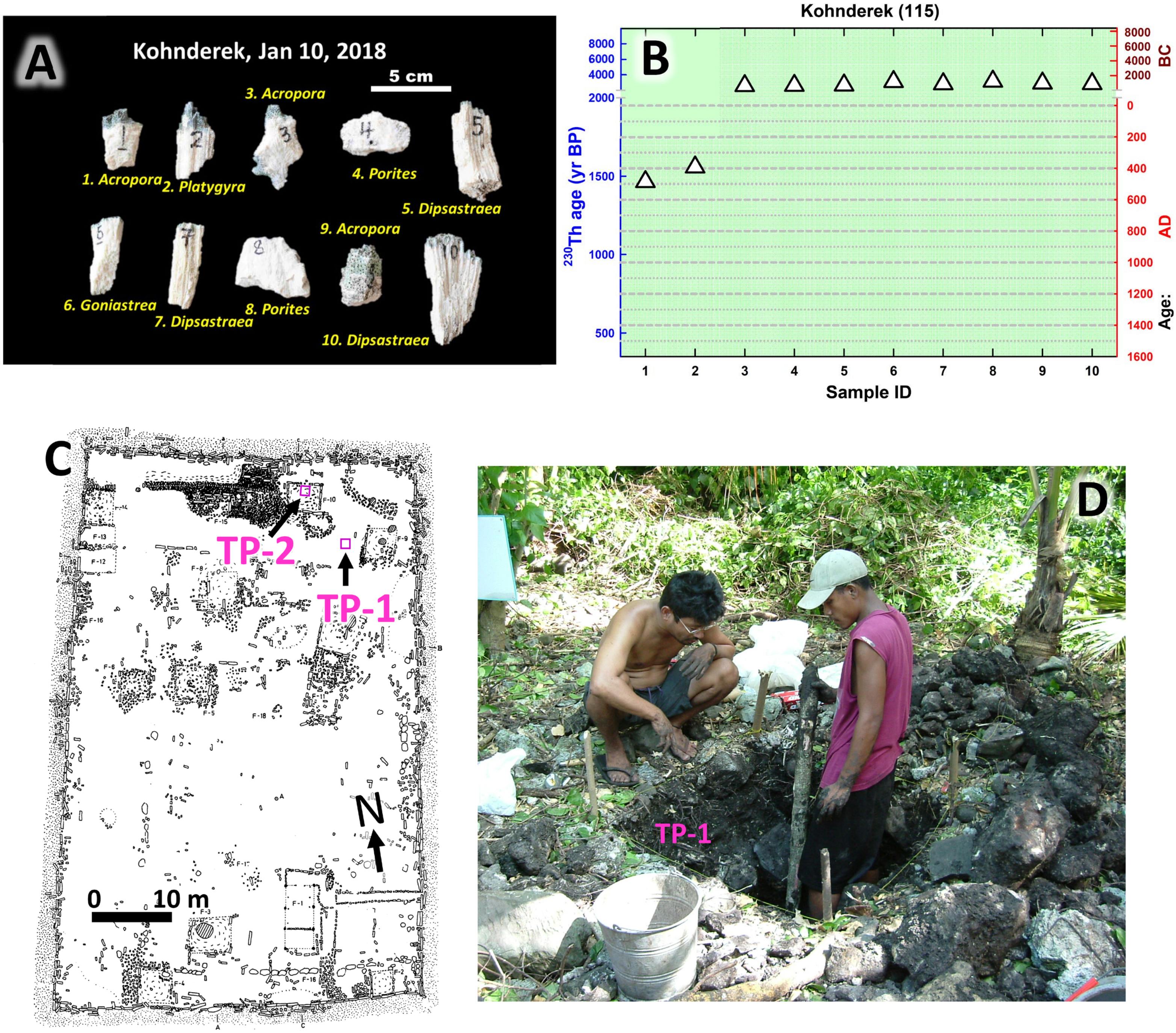

Fig. S6. Corals and charcoals collected from Kohnderek Islet. (A) Ten coral subsamples were collected in 2018. **(B)** The determined ^{230}Th ages. **(C)** Two test pits, 1x1 m, were excavated on Kohnderek (map modified from ref. 63). Charcoal samples from both TP-1 and TP-2 were collected during August 17-20, 2005, for ^{14}C dating (Data S2). **(D)** A photograph of test pit TP-1 (taken by Kataoka in 2005; courtesy of the National Museum of Ethnology, Osaka, Japan, used with permission).

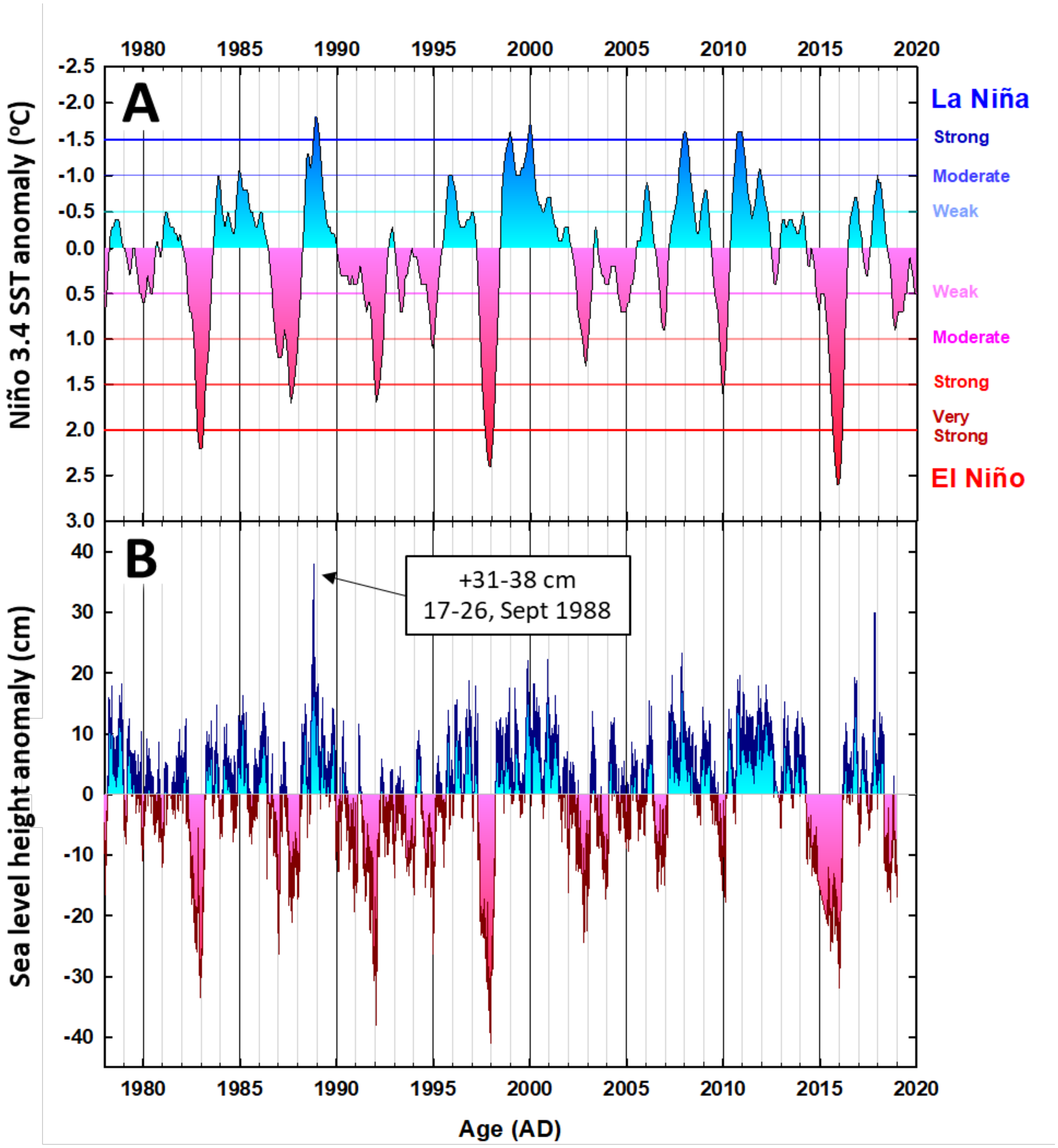

Fig. S7. Instrumental records of El Niño-Southern Oscillation (ENSO) index and Pohnpei daily sea level height anomaly from CE 1978-2020. (A) Time series of monthly Niño 3.4 (5N-5S, 170W-120W) sea surface temperature (SST) anomaly, relative to a base of 30 years (CE 1971-2000). (Data from National Center for Atmospheric Research, https://climatedataguide.ucar.edu/climate-data/nino-sst-indices-nino-12-3-34-4-oni-and-tni). **(B)** Pohnpei daily sea level height anomaly record, corrected for global sea-level change[64]. (Data from University of Hawaii, Sea Level Center, https://uhslc.soest.hawaii.edu/).

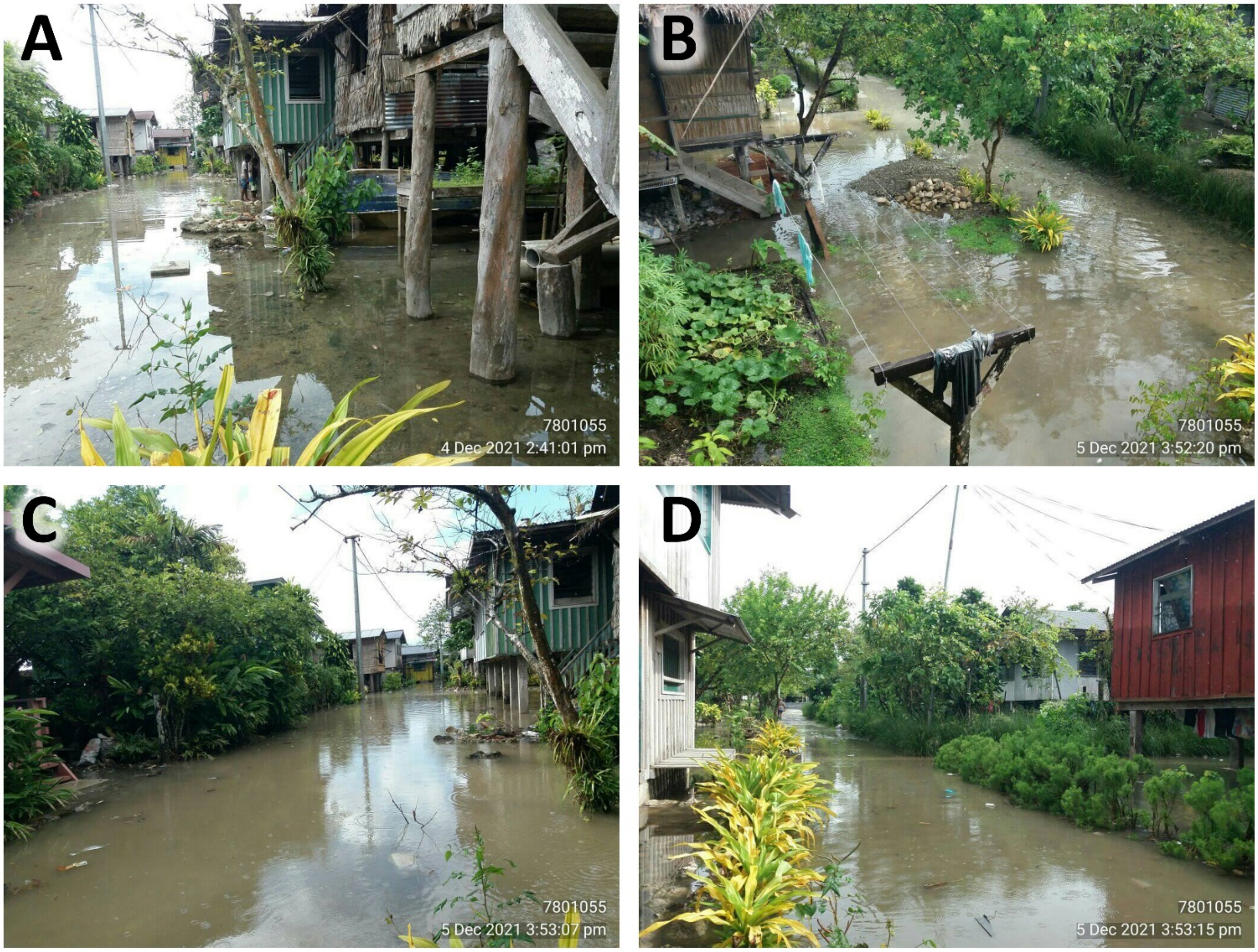

Fig. S8. Seawater intrusion to villages (A-D) of Auki, Malaita, Solomon Islands on December 4-5, 2021. (Courtesy of Timmy Saki Misimake, used with permission.)

References

63. Athens, S. J. *Archaeological Investigations at Nan Madol: Islet Maps and Surface Artifacts*. Pacific Studies Institute Monograph Series, 2. Guam: Pacific Studies Institute (1980).

64. Frederikse, T. et al. The causes of sea-level rise since 1900. *Nature* **584**, 393-397 (2020).
